# Case Report: *TRPV4* gene mutation causing neuronopathy, distal hereditary motor, type VIII

**DOI:** 10.3389/fped.2024.1327742

**Published:** 2024-03-18

**Authors:** Fengge Wang, Xuemei Jin, Yongning Zhu, Shuli Jiang, Xiaoyan Zhang, Yanping Wang, Dongmei Man, Fuling Wang

**Affiliations:** Department of Obstetrics, Affiliated Hospital of Jining Medical University, Jining Medical University, Jining, China

**Keywords:** neuronopathy, distal hereditary motor, type VIII, *TRPV4*, gene mutation, c.805C>T, p.Arg269Cys, bilateral clubfoot

## Abstract

Neuronopathy, distal hereditary motor, type VIII is an exceedingly rare autosomal dominant genetic disorder, also known as congenital non-progressive distal spinal muscular atrophy. It is characterized by progressive weakness in distal motor function and atrophy of muscles, without accompanying sensory impairment. Presently, there is limited literature on this condition, and accurate epidemiological data regarding its incidence remains unavailable. We report a paediatric case of distal hereditary motor, type VIII that is caused by a heterozygous missense mutation in the *TRPV4* gene (NM_021625): c.805C>T. The proband is a 7-year-old male child. During pregnancy, his mother had prenatal ultrasound revealing “inward turning of the feet”, a condition persisting after birth. The proband is currently unable to stand independently, exhibiting bilateral clubfoot deformity. Although possessing normal cognitive function, he cannot walk unaided. Computed radiography findings reveal pelvic tilt, bilateral knee joint valgus, and bilateral clubfoot. The patient underwent familial exome sequencing, revealing a mutation in the *TRPV4* gene (NM_021625): c.805C>T (p.Arg269Cys). Considering the patient’s medical history, clinical manifestations, imaging studies, and genetic test results, the diagnosis for this individual is Neuronopathy, distal hereditary motor, type VIII. This report documents a case involving the *TRPV4* gene mutation associated with Neuronopathy, distal hereditary motor, type VIII, contributing valuable case reference for the early diagnosis of this condition.

## 1 Introduction

Neuronopathy, distal hereditary motor (OMIM 600175), also known as congenital neuropathy, distal hereditary motor (NDHM), constitutes a heterogeneous group of neuro-muscular disorders caused by anterior horn cell degeneration. Characterized by progressive weakness in distal motor function and distal muscular atrophy. NDHM presents without sensory impairment (1–3). The seven subtypes of NDHM are classified based on their genetic patterns and phenotypes. *TRPV4*, also known as transient receptor potential vanilloid 4, is located on chromosome 12q24.1 and comprises 16 exons (4–6). A fair amount of genotype-phenotype variability exists between subjects carrying the same TRPV4 mutation (7). Dominant mutations in *TRPV4* have been described in both peripheral nervous system and skeletal diseases. The *TRPV4* gene mutation in this case is associated with NDHM VIII. In addition, changes in the *TRPV4* gene may cause other disorders including scapuloperoneal spinal muscular atrophy and Charcot-Marie-Tooth disease type 2C. NDHM VIII is inherited in an autosomal dominant manner and primarily affects the lower limbs (more distal than proximal) (7, 8). Typically congenital, clinical manifestations encompass non-progressive muscle atrophy, thigh muscle wasting, weakness in the adductors of the thigh, weakness in the extensors of the knee and ankle, weakness in the smallest jaw muscle and flexors of the neck, contractures in the knee joint and hip flexion, and severe bilateral clubfoot (9, 10). The rarity of this disease is underscored by limited global literature on the subject.

## 2 Case report

**Figure 1:**
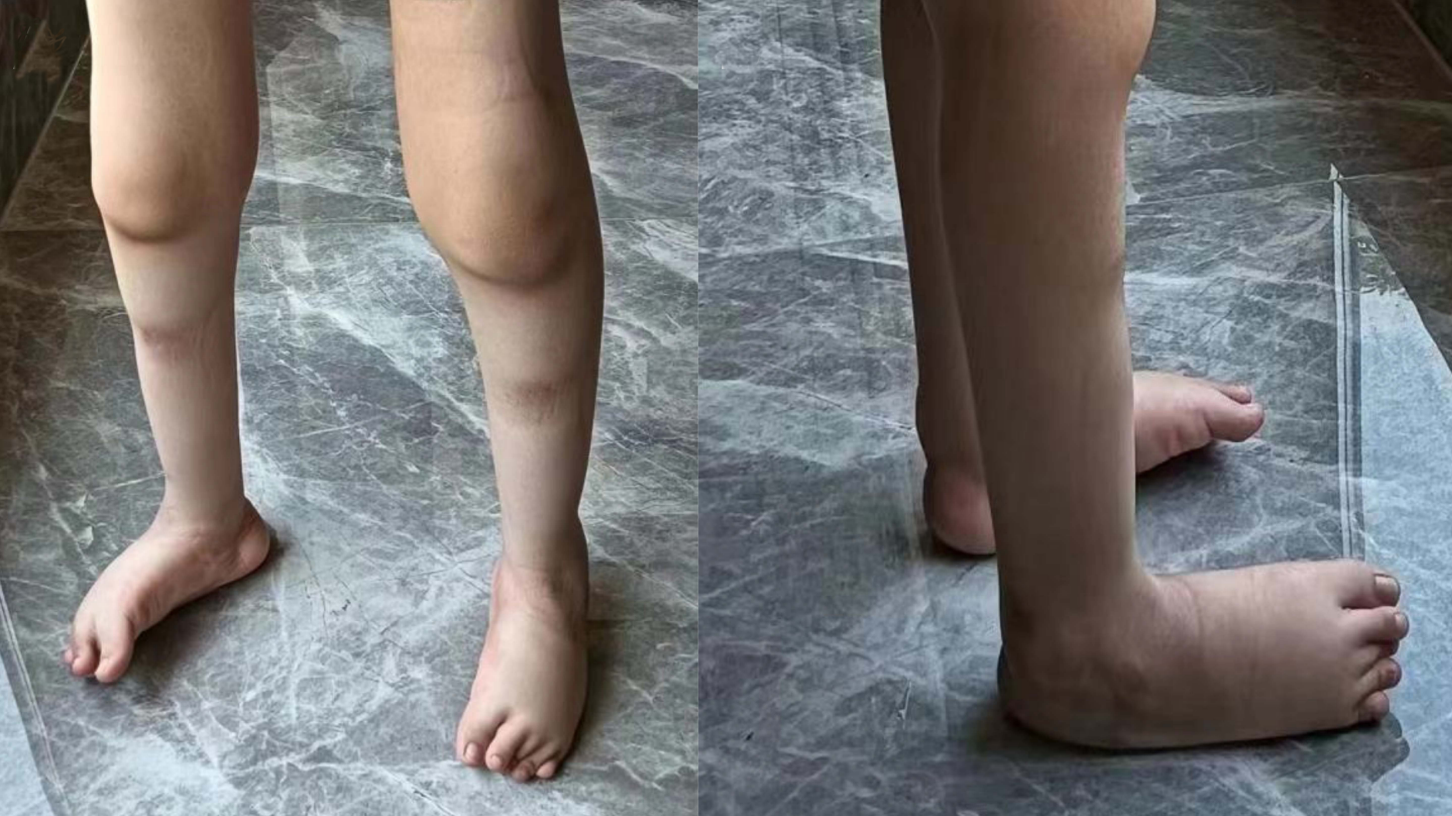
Imaging of the affected lower limbs in the proband. The primary clinical manifestations in the patient manifest as clubfoot and distal muscle atrophy in the legs.

The patient (proband) is a 7-year-old male, weighing 25 kg, born in 2016, who sought consultation at our genetics clinic due to the inability to walk independently and bilateral clubfoot persisting at the age of 7. During pregnancy, the patient’s mother had prenatal ultrasound revealing “inward turning of the feet”. Postnatally, the lower limbs exhibited deformities with symptomatic clubfoot (Figure 1). At present, the patient is 7 years old, still unable to walk independently, with normal intelligence but diminished attention span. Muscular atrophy is observed bilaterally in the lower limbs, particularly evident in the quadriceps and calf muscles, with significant atrophy in the muscles surrounding the knee joint. Passive extension of the knee joint is restricted, and clubfoot deformity and muscle softness are evident in the feet. Muscle strength in both lower limbs is graded between 3 and 4. The left lower limb exhibits comparatively weaker muscle strength, enabling crawling but limiting the ability to stand on one knee, and transitioning from single-knee to standing is unattainable. Walking with corrective shoes results in internal rotation of both lower limbs, particularly pronounced on the right side. Walking is unstable, prone to falling, but the patient can rise directly from the ground with the support of both hands after a fall. Wearing corrective shoes, the patient cannot clear obstacles higher than 20 cm, lift both feet off the ground, and laboratory tests including urine routines, blood routine examination, liver and renal function and c-reactive protein testing reveal no abnormalities. Currently, paresis of the vocal cord has been excluded by laryngoscopy. The phenotypes of the patient’s parents are unremarkable, with no consanguinity, and no reported family history of related diseases.

**Figure 2:**
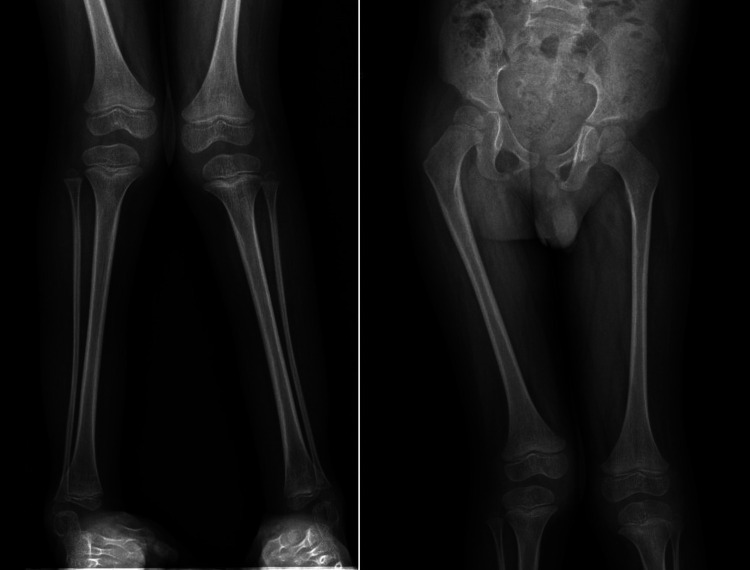
Computed radiography (CR) images of the proband's lower limbs. The patient's lower limbs exhibit pelvic tilt, bilateral knee joint valgus, and bilateral clubfoot.

**Figure 3:**
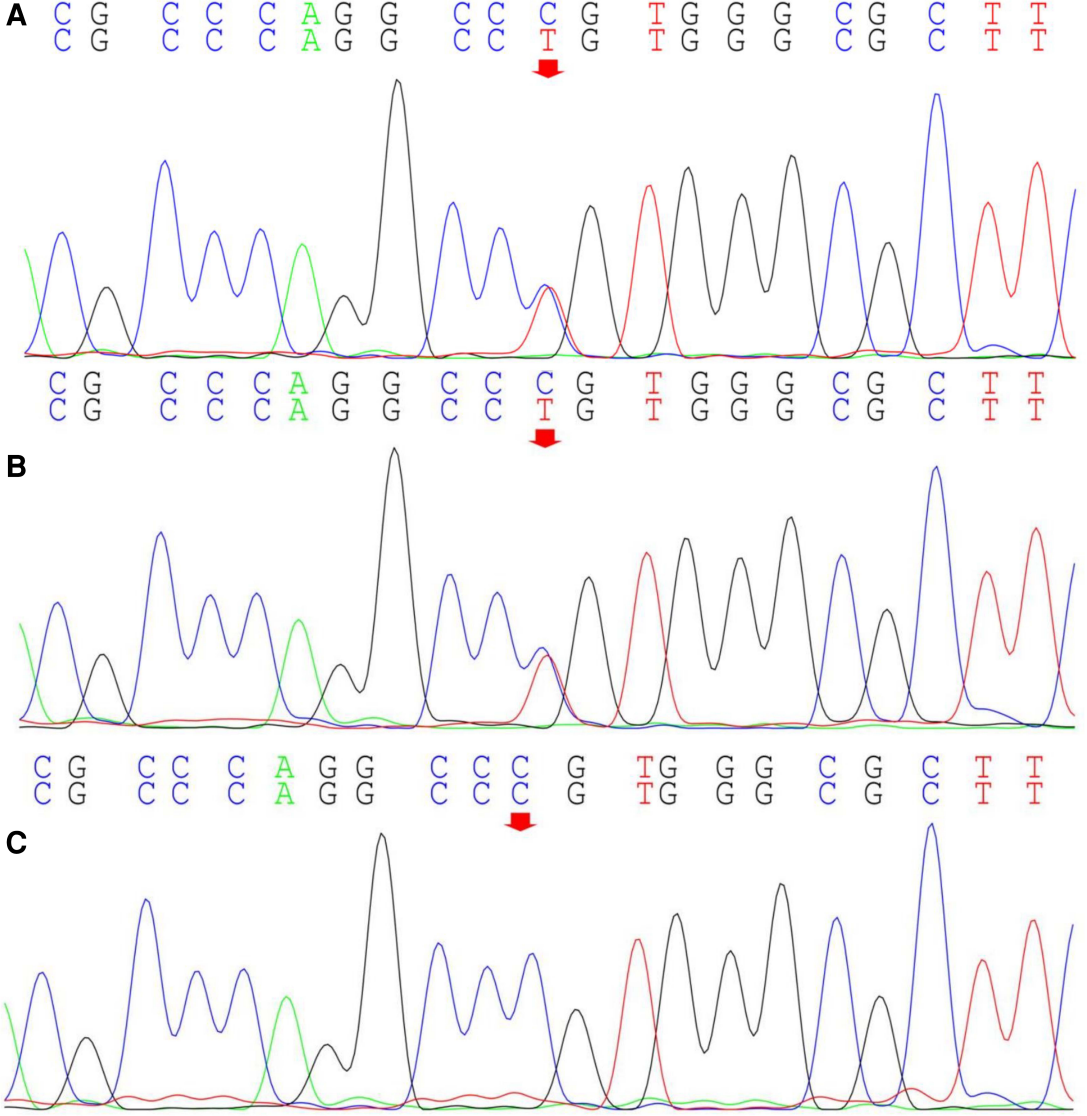
Sanger sequencing of the *TRPV4* gene locus in the proband and the proband's parents. The proband (**A**) exhibits a heterozygous mutation in the *TRPV4* gene at position c.805C>T (indicated by the arrow), inherited from the proband's father (**B**) (indicated by the arrow); The proband's mother does not harbor this mutation (**C**).

Computed radiography (CR) images of the patient's lower limbs exhibit pelvic obliquity, bilateral knee joint valgus, and bilateral clubfoot. The bone structures of the bilateral femurs, tibias and fibulas, as well as the hip joints, showed no apparent abnormalities (Figure 2). To ascertain the diagnosis, familial exome sequencing was conducted with informed consent from the patient and his parents. The results indicated a heterozygous missense mutation at position 805 (c.805C>T) in the 5th exon of the *TRPV4* gene, leading to a substitution of arginine at position 269 with cysteine. The ACMG classification for this variant is pathogenic. This mutation was inherited from the father, who, despite being phenotypically asymptomatic, is presumed to be a non-manifesting carrier. To validate this finding, Sanger sequencing was performed, confirming once again that the mutation was inherited from the father (Figure 3).

Combining the patient's medical history, clinical manifestations, imaging studies, and genetic test results, the diagnosis for this individual is Neuronopathy, distal hereditary motor, type VIII (OMIM: 600175) (11). Neuronopathy, distal hereditary motor, type VIII is an autosomal dominant genetic disorder, representing a rare form of distal hereditary motor neuropathy. The clinical phenotype is variable, with characteristic features including congenital, non-progressive weakness and atrophy primarily affecting the distal muscles of the lower limbs, as well as congenital (or early-onset) contractures of the hip, knee, and ankle joints. Additional manifestations may include reduced or absent deep tendon reflexes in the lower limbs, skeletal abnormalities (bilateral clubfoot, scoliosis, kyphosis, lordosis, and increased lumbar lordosis), delayed walking, unsteady gait, joint hypermobility, and commonly associated bladder and bowel dysfunction. In addition, muscle MRI analysis of the patient's father revealed no significant abnormalities in the morphology and signals of the bilateral thigh muscle groups (Supplementary Figure S1). Electrophysiological examination of the patient's father revealed no significant abnormalities in resting potentials, duration, amplitude, and recruitment phase (Supplementary Table S1). Taking everything into account, it is plausible that the patient's father may be a non-manifesting carrier or has symptoms too mild to be identified.

## 3 Discussion

Neuronopathy, distal hereditary motor, type VIII is a rare genetic disorder. Affected individuals typically exhibit symptoms between birth and 25 years of age (7). In this case, the prenatal ultrasound of the patient's mother revealed “inward turning of the feet”. Postnatally, the lower limbs exhibited deformities with symptomatic clubfoot. Moreover, the clinical manifestations for NDHM VIII vary greatly (7, 12). Individuals with mild involvement may solely manifest congenital weakness in the distal lower limbs, while severely affected individuals may present with weakness in the pelvic girdle and trunk muscles, resulting in scoliosis and diminished or absent reflexes (13). Astrea et al. reported two children with NDHM VIII who exhibited similar clinical symptoms to the patient in this case, such as proximal and distal muscle weakness, distal muscle atrophy in the legs, and clubfoot. CR imaging revealed extensive fat atrophy in the thigh muscles, preservation of the lateral aspect of the quadriceps in the thighs, and preservation of the posterior medial aspect of the calf muscles. In addition, the patient in this case exhibited symptoms of pelvic obliquity and bilateral knee joint valgus. TRPV4-related motor axonal neuropathy is frequently associated with vocal cord paralysis (8). Also, some patients with NDHM VIII will exhibit symptoms of paresis of the vocal cord. To date, the patient in this case has not exhibited paresis of the vocal cord.

Additionally, in some cases, clinical symptoms may manifest subtly, to the extent that affected individuals may go unnoticed (14). Furthermore, disease penetrance resulting from *TRPV4* mutations is incomplete (9), and there are numerous reports of incomplete penetrance in TRPV4-related diseases. For instance, one study reported a family with five individuals carrying the *TRPV4* gene (NM_021625): c.805C>T mutation, with varying phenotypes. A 44-year-old female exhibited scapuloperoneal spinal muscular atrophy, while the proband's 7-year-old daughter suffered from NDHM VIII. The daughter presented with severe congenital spinal muscular atrophy with arthrogryposis, laryngomalacia, and vocal cord paresis (14). The other three mutation carriers, aged 9, 40, and 70, showed no clinical symptoms, indicating incomplete penetrance (14). Additionally, Echaniz-Laguna et al. reported members from twelve families with pathogenic mutations in *TRPV4*, but only seven displayed clinical phenotypes and were diagnosed with NDHM VIII, with evidence of incomplete penetrance in two families (7). In this case, the *TRPV4* (NM_021625): c.805C>T mutation in the child is inherited from the father. The primary pathological phenotype in the child includes bilateral clubfoot, distal muscle weakness and atrophy in the lower limbs, pelvic tilt, bilateral knee joint valgus, and the inability to stand and walk independently. However, the father exhibits no abnormal phenotype. Furthermore, muscle MRI analysis and electrophysiologic characteristics of the patient's father revealed no significant abnormalities. Considering all the evidence, it is plausible that the father may be a non-manifesting carrier or has symptoms too mild to be identified.

Functioning as a Ca^2+^-permeable, non-selective cation channel, TRPV4 is sensitive to physical, hormonal, and chemical stimuli. It is expressed in various cell types, including osteoclasts, chondrocytes, and sensory neurons (5). Presently, the vast majority of studies have suggested that a gain-of-function mechanism is responsible for the TRPV4-associated neuropathies due to cytotoxicity from increased intracellular calcium concentration (15–17). This likely constitutes the primary pathogenic mechanism underlying NDHM VIII. The p.Arg269Cys mutation in this case occurs in the fifth exon of TRPV4 and represents a missense mutation. It has been reported that missense mutations in *TRPV4* disrupt the normal transport of intracellular and extracellular calcium ions, resulting in elevated cytoplasmic Ca^2+^ levels and exacerbating cellular toxicity, contributing to disease onset (18). Conversely, a few studies on the molecular pathogenesis of *TRPV4* mutations in NDHM VIII are centered on haploinsufficiency (10).

NDHM VIII is a rare genetic disorder for which there is currently no specific treatment. At present, the focus of treatment is on symptom management. Main treatment modalities include physical rehabilitation therapy, orthoses and assistive devices, as well as occupational therapy (19). Due to the rarity of the condition, there is limited post-treatment reporting. A case report described a male patient with NDHM VIII, presenting with congenital foot deformities and a severe clinical phenotype of spinal lordosis from birth. Despite undergoing multiple corrective surgeries postnatally, the prognosis remained poor, with no significant improvement in the abnormal clinical phenotype, and the emergence of a phenotype featuring bony ankylosis of the hip, knee, and ankle joints (11). Neurological examination revealed weakness in both legs, paralysis of the dorsiflexors of both feet, muscle atrophy, and the absence of muscle stretch reflexes in the legs. The proband is currently undergoing physical rehabilitation therapy (11).

As previously discussed, NDHM VIII, caused by *TRPV4*, is inherited in an autosomal dominant manner, with the majority of individuals diagnosed with this condition likely inheriting pathogenic mutations from their parents (20). In this case, the patient also inherited the pathogenic mutation from his father who may be a non-manifesting carrier or has symptoms too mild to be identified. If the parents of the proband do not carry the *TRPV4* pathogenic variant, there exists the theoretical possibility of the parents being germline mosaics. Due to reduced penetrance and variability in expressivity, predicting specific phenotypes, age of onset, and disease severity accurately is challenging. However, generally, children inheriting *TRPV4* pathogenic variants associated with NDHM VIII from affected parents may exhibit similar phenotypes to their parents (21). In families with affected members carrying relevant pathogenic variants, pre-implantation genetic testing and prenatal diagnosis can be considered.

## 4 Conclusion

In conclusion, genetic testing plays a crucial role in the diagnosis of NDHM VIII, aiding in unraveling its intricate molecular pathogenesis. The ongoing development of scientific research and the increasing awareness of rare diseases are poised to make prevention and treatment of NDHM VIII possible. It is anticipated that in the future, efforts will be directed towards enhancing the screening of common pathogenic genes in patients with NDHM VIII, increasing the rate of pre-symptomatic and prenatal diagnoses, and facilitating the optimization of treatment methods.

## Data Availability

The original contributions presented in the study are included in the article/**Supplementary Material**, further inquiries can be directed to the corresponding authors.

## Supplementary material

Supplementary Figure S1 T1-weighted magnetic resonance images of the thighs and lower legs of the proband's father. (**A**) Axial T1-weighted MRI image of thighs; (**B**) anterior coronal MRI image of thighs; (**C**) axial T1-weighted MRI image of lower leg; (**D**) anterior coronal MRI image of lower legs.
