# Supplementary material for: Case Report: TRPV4 gene mutation causing neuronopathy, distal hereditary motor, type VIII
Source: Front Pediatr. 2024 Mar 18;12:1327742. doi: 10.3389/fped.2024.1327742 (PMC10982358; doi:10.3389/fped.2024.1327742)
Supplement: Supplementary Figure S1 — T1-weighted magnetic resonance images of the thighs and lower legs of the proband's father. (A) Axial T1-weighted MRI image of thighs; (B) anterior coronal MRI image of thighs; (C) axial T1-weighted MRI image of lower leg; (D) anterior coronal MRI image of lower legs. [file Table1.docx]

**Table S1:Electromyogram (EMG) studies of patient’s father**

| **Spontaneous potential** | **Average duration**  **ms** | **Average amplitude**  **uV** | **%Poiy**  **%** | **Maximal contraction waveform** |
| --- | --- | --- | --- | --- |
| **Left**  **Tibialis anterior** | 10.9 | 1333 | 66.7 | mixed phase |
| **Right**  **Tibialis anterior** | 10.2 | 1188 | 0 | mixed phase |
| **Left**  **Vastus medialis** | 10.6 | 616 | 0 | mixed phase |
| **Right**  **Vastus medialis** | 11.5 | 1302 | 0 | mixed phase |
| **Left**  **Gastrocnemius (medial head)** | 9.4 | 1006 | 100 | mixed phase |
| **Right**  **Gastrocnemius (medial head)** | 10.2 | 684 | 0 | mixed phase |
